# Identification of Potential Therapeutic Targets and Immune Cell Infiltration Characteristics in Osteosarcoma Using Bioinformatics Strategy

**DOI:** 10.3389/fonc.2020.01628

**Published:** 2020-08-21

**Authors:** Jianfang Niu, Taiqiang Yan, Wei Guo, Wei Wang, Zhiqing Zhao, Tingting Ren, Yi Huang, Hongliang Zhang, Yiyang Yu, Xin Liang

**Affiliations:** ^1^Musculoskeletal Tumor Center, Peking University People’s Hospital, Peking University, Beijing, China; ^2^Beijing Key Laboratory of Musculoskeletal Tumor, Peking University People’s Hospital, Beijing, China

**Keywords:** bioinformatics analysis, osteosarcoma, robust rank aggregation, immune cell infiltration, survival analysis

## Abstract

Osteosarcoma is one of the most aggressive malignant bone tumors worldwide. Although great advancements have been made in its treatment owing to the advent of neoadjuvant chemotherapy, the problem of lung metastasis is a major obstacle in the improvement of survival outcomes. Thus, the aim of the present study is to screen novel and key biomarkers, which may act as potential prognostic markers and therapeutic targets in osteosarcoma. We utilized the robust rank aggregation (RRA) method to integrate three osteosarcoma microarray datasets downloaded from the Gene Expression Omnibus (GEO) database, and we identified the robust differentially expressed genes (DEGs) between primary and metastatic osteosarcoma tissues. Gene Ontology (GO) and Kyoto Encyclopedia of Genes and Genomes (KEGG) enrichment analyses were performed to explore the functions of robust DEGs. The results of enrichment analysis showed that the robust DEGs were closely associated with osteosarcoma development and progression. Immune cell infiltration analysis was also conducted by CIBERSORT algorithm, and we found that macrophages are the most principal infiltrating immune cells in osteosarcoma, especially macrophages M0 and M2. Then, the protein–protein interaction network and key modules were constructed by Cytoscape, and 10 hub genes were selected by plugin cytoHubba from the whole network. The survival analysis of hub genes was also carried out based on the Therapeutically Applicable Research to Generate Effective Treatments (TARGET) database. The integrated bioinformatics analysis was utilized to provide new insight into osteosarcoma development and metastasis and identified *EGR1*, *CXCL10*, *MYC*, and *CXCR4* as potential biomarkers for prognosis of osteosarcoma.

## Introduction

Osteosarcoma is one of the most aggressive malignant tumors in the bone (1), which derived from mesenchymal tissue and showed osteoblastic differentiation (2). Annually, the incidence rate of osteosarcoma is approximately four to five cases per million (3). In addition, osteosarcoma is most prevalent in children and adolescents (4), and 15–20% of osteosarcoma patients have lung metastasis at the initial diagnosis (5, 6). With the assistance of neoadjuvant chemotherapy, the treatment of osteosarcoma has been greatly improved, but the overall survival of patients with lung metastasis or relapse has not improved and remains low at approximately 20% (7, 8). Therefore, it is extremely necessary to seek novel prognostic factors and therapeutic targets for osteosarcoma.

In recent years, public databases including Gene Expression Omnibus (GEO), The Cancer Genome Atlas (TCGA), and Therapeutically Applicable Research to Generate Effective Treatments (TARGET) are widely used to explore diagnostic and prognostic biomarkers in osteosarcoma. In the previous studies, the limited number of samples and inappropriate analysis methods of multiple datasets led to deviation of the results. Zhang et al. (9) and Diao et al. (10) described the role of gene copy number alterations and methylation changes in the malignant progression of osteosarcoma using bioinformatics analyses, respectively. However, these studies were only based on single datasets and had a limited sample size, which may have biased the final results. To analyze more samples and avoid the sample heterogeneity of each independent experiment and the error caused by different technology platforms and different data processing methods, we used the robust rank aggregation (RRA) method to obtain robust differentially expressed genes (DEGs). RRA was used to compare the ranking of multiple gene lists. If a gene ranked the highest in all gene lists, then the smaller its calculated *P*-value is, the more likely it is to be a robust DEG (11). This method has been widely used in integrated analysis of multiple datasets, and it is robust to errors and noise (12–14). There have been no reports of the use of RRA in osteosarcoma.

In our study, microarray datasets GSE14827, GSE21257, and GSE32981 from GEO database were downloaded and analyzed by RRA method to identify robust DEGs between primary and metastatic osteosarcoma tissues. A total of 524 robust DEGs were determined, including 272 upregulated and 252 downregulated genes. Gene Ontology (GO) and Kyoto Encyclopedia of Genes and Genomes (KEGG) enrichment analyses were performed to explore the functions of robust DEGs. Immune cell infiltration analysis was also conducted by CIBERSORT algorithm. In addition, we also constructed the protein–protein interaction (PPI) network and key modules, and finally 10 hub genes were selected by plugin cytoHubba from the whole network. Survival analysis of hub genes was carried out using R packages. In conclusion, the integrated bioinformatics analysis was utilized to identify the significant robust DEGs and hub genes, which may act as novel and potential prognostic biomarkers in osteosarcoma.

## Materials and Methods

### Data Collection and Data Processing

We selected three gene chips of osteosarcoma from GEO database^1^, including GSE14827, GSE21257, and GSE32981. The selection criteria were as follows: (1) inclusion of primary and metastatic osteosarcoma tissue samples; (2) expression profiling by array as the experiment type; (3) *Homo sapiens*; and (4) 20 samples as the minimal size. Among them, GSE14827 contained 18 primary samples and 9 metastatic osteosarcoma samples. GSE21257 contained 19 non-metastatic and 34 metastatic osteosarcoma samples. The GSE32981 dataset contained 23 samples. One sample was not available and was excluded. Twenty-two samples were selected for further study including four primary and 18 metastatic samples. The matrix files and platform annotation document of three microarray datasets were downloaded. The names of microarray probes were converted to the gene symbols by Perl. The DEGs were identified between primary and metastatic osteosarcoma samples in each dataset by limma package in R (15) with the cutoff criteria of |log2 fold change (FC)| > 0.585 and *P*-value < 0.05.

### Robust Rank Aggregation Analysis

To integrate the three microarray datasets, RRA method was used to determine the robust DEGs (16), which is a standard method to minimize the bias and errors among several datasets. Before RRA analysis, the upregulated and downregulated genes were ranked by their FC in each dataset. Then, the RobustRankAggreg R package was performed to get robust DEGs on the basis of the ranked genes in the three datasets. Genes with FC > 1.3 and *P*-value < 0.05 were considered as the significant robust DEGs.

### Gene Ontology Function and Kyoto Encyclopedia of Genes and Genomes Pathway Enrichment Analyses

To identify the functional roles of the robust DEGs indicated above, GO enrichment results of biological process (BP), cellular component (CC), and molecular function (MF) were obtained using the R package “clusterprofiler.” The KEGG pathway analysis of robust DEGs was also conducted using the R package (17). *P* < 0.05 was considered statistically significant.

### Immune Infiltration by CIBERSORT Analysis

The CIBERSORT algorithm is commonly used to predict the infiltration of 22 types of immune cells in each tissue sample (18). The 22 kinds of immune cells include seven types of T cells [CD8^+^ T cells, naïve CD4^+^ T cells, resting memory CD4^+^ T cells, activated memory CD4^+^ T cells, follicular helper T cells, regulatory T cells (Tregs), gamma delta T cells], three types of macrophages (M0, M1, and M2), naïve B cells, memory B cells, plasma cells, resting natural killer (NK) cells, activated NK cells, monocytes, resting dendritic cells, activated dendritic cells, resting mast cells, activated mast cells, eosinophils, and neutrophils. Normalized gene expression matrix was converted to 22 types of immune cell matrix by the CIBERSORT algorithm. The immune cell matrix was filtered according to the criteria of *P* < 0.05, and then the relative expression of 22 types of immune cells was identified between primary and metastatic osteosarcoma samples by R packages. The principal component analysis (PCA) was also performed to determine the difference between primary and metastatic samples.

### Protein–Protein Interaction Network Construction and Module Analysis

We uploaded the robust DEGs to the STRING online database^2^, and we chose confidence >0.9 as the screening criteria. The visualized PPI network was performed by Cytoscape (version 3.6.1) software^3^. Cytoscape plugin-MCODE was used to screen the significant modules in the PPI network.

### Hub Gene Identification

cytoHubba, a plugin of Cytoscape, provides several topological analysis algorithms, including Degree, Edge Percolated Component (EPC), Maximum Neighborhood Component (MNC), Density of Maximum Neighborhood Component (DMNC), Maximal Clique Centrality (MCC), and six centralities that include BottleNeck, EcCentricity, Closeness, Radiality, Betweenness, and Stress. These algorithms can be used to identify hub genes (19).

### Survival Analysis

The RNA-seq-FPKM data and prognostic information of osteosarcoma patients were downloaded from TARGET database^4^. TARGET is a database that only includes children’s tumors. Presently, TARGET database contains six kinds of tumors, including ALL (Acute Lymphoblastic Leukemia), AML (Acute Myeloid Leukemia), KT (Kidney Tumors), MDLS (Model Systems), NBL (Neuroblastoma), and OS (Osteosarcoma). The survival analyses of hub genes were conducted by R package survival and survminer. *P* < 0.05 was considered to be statistically significant.

## Results

### Identification of Differentially Expressed Genes in Each Dataset

**FIGURE 1:**
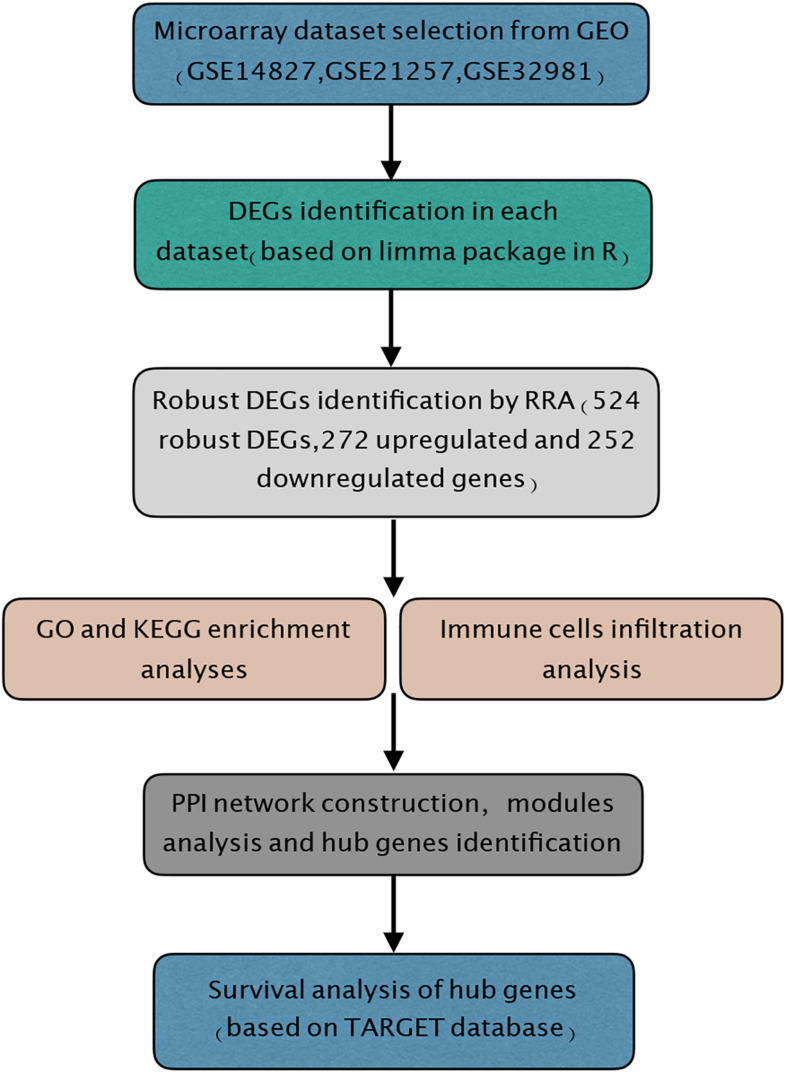
Study workflow. GEO, Gene Expression Omnibus; GO, Gene Ontology; KEGG, Kyoto Encyclopedia of Genes and Genomes; RRA, robust rank aggregation; TARGET, Therapeutically Applicable Research to Generate Effective Treatments.

**FIGURE 2:**
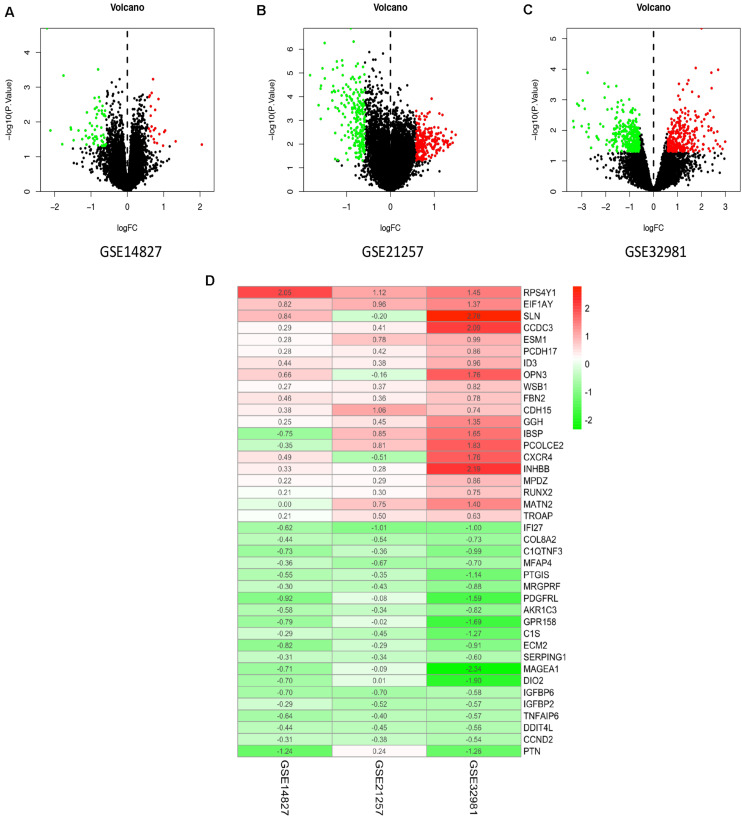
Identification of DEGs and robust DEGs. Volcano plots of the distribution of DEGs in GSE14827 **(A)**, GSE21257 **(B)**, and GSE32981 **(C)**. Red and green dots represent the upregulated and downregulated genes, respectively. **(D)** The heatmap of top 20 upregulated and downregulated robust DEGs identified by RRA method. Red represents high expression robust DEGs, while blue represents low expression robust DEGs. DEG, differentially expressed gene; RRA, robust rank aggregation.

In the present study, the biological characteristics of DEGs were identified by integrated bioinformatics analysis. The overall workflow of this study is showed in Figure 1. The osteosarcoma microarray data GSE14827, GSE21257, and GSE32981 were selected and analyzed using limma package in R. A total of 102 osteosarcoma samples including 41 primary and 61 metastatic tissues were identified in our study. According to the cutoff criteria of | log2 FC| > 0.585 and *P* < 0.05, there were 83 DEGs in GSE14827 including 23 upregulated and 60 downregulated genes. A total of 464 DEGs were screened from the GSE21257 dataset, including 247 upregulated and 217 downregulated genes. Additionally, a total of 650 DEGs were selected in GSE32981, including 299 upregulated and 351 downregulated genes. The distribution of DEGs is shown in volcano plots (Figures 2A–C), where the red and green dots represent the upregulated and downregulated genes, respectively.

### Identification of Robust Differentially Expressed Genes by Robust Rank Aggregation Method

To integrate the three datasets with minimal bias, the RRA method was used. A total of 524 robust DEGs were determined, including 272 upregulated and 252 downregulated genes (Supplementary Table S1). According to the *P*-value of robust DEGs, we assigned the top 20 upregulated and downregulated robust DEGs in the visualized heatmap (Figure 2D).

### Functional Enrichment Analyses of Robust Differentially Expressed Genes

**FIGURE 3:**
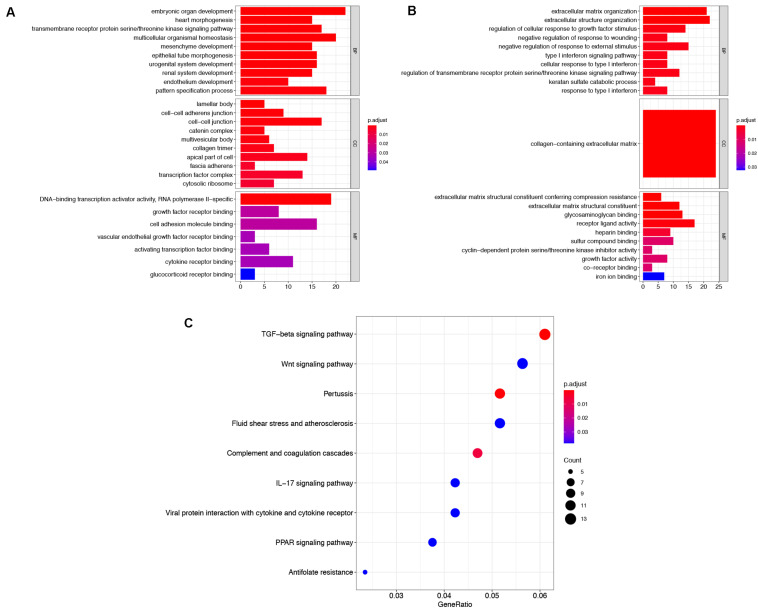
Functional enrichment analysis of robust DEGs. GO enrichment analyses of upregulated robust DEGs **(A)** and downregulated robust DEGs **(B)** in three parts: BP, CC, and MF. **(C)** KEGG pathway enrichment analysis of robust DEGs. DEG, differentially expressed gene; GO, Gene Ontology; BP, biological process; CC, cellular component; MF, molecular function; KEGG, Kyoto Encyclopedia of Genes and Genomes.

To explore the functions of robust DEGs, the GO and KEGG enrichment analyses were conducted by R packages. The results of GO analysis included three categories: BP, CC, and MF. For BP, the upregulated robust DEGs were mainly enriched in embryonic organ development, multicellular organismal homeostasis, and transmembrane receptor protein serine/threonine kinase signaling pathway. In the CC part, the upregulated genes were particularly enriched in lamellar body, cell–cell junction, and cell–cell adherens junction. The top three significantly enriched terms were DNA-binding transcription activator activity, growth factor receptor binding, and cell adhesion molecule binding in the MF group (Figure 3A and Supplementary Table S2). Moreover, the most significantly enriched GO BP terms of downregulated genes were extracellular matrix (ECM) organization, extracellular structure organization, and regulation of cellular response to growth factor stimulus. For CC category, the downregulated genes were enriched in collagen-containing ECM. In addition, the downregulated DEGs were mainly enriched in ECM structural constituent conferring compression resistance, ECM structural constituent, and glycosaminoglycan binding in the MF group (Figure 3B and Supplementary Table S3). The above results indicated that the robust DEGs were mostly associated with cancer-related functions.

The result of KEGG pathway enrichment analysis is also shown in Figure 3C. TGF-beta signaling pathway, wnt signaling pathway, and IL-17 signaling pathway were highly associated with tumor progression.

### Immune Cell Infiltration Analysis

**FIGURE 4:**
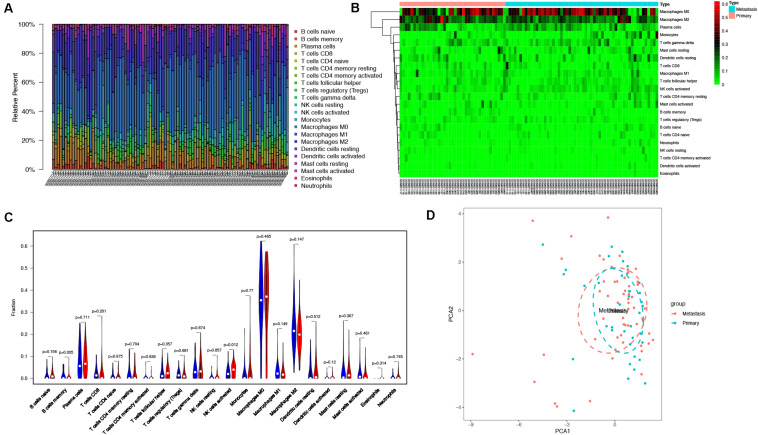
Immune cells infiltration analysis. **(A)** The distribution of 22 types of immune cells between primary and metastatic osteosarcoma tissues. **(B)** The difference of immune cells infiltration between primary and metastatic osteosarcoma tissues visualized by heatmap. **(C)** Violin plot visualizing the differentially infiltrated immune cells (*P* < 0.05). **(D)** PCA performed on all osteosarcoma tissues. The two principal components showed nothing significant variation. PCA, principal component analysis.

With the use of CIBERSORT algorithm, the infiltration of 22 kinds of immune cells in 102 osteosarcoma tissues is shown in Figure 4A. There was no significant difference in the infiltration of immune cells between primary and metastatic osteosarcoma tissues. However, compared with other immune cells, such as T cells and B cells, macrophage infiltration dominated, whether in primary or metastatic osteosarcoma tissues (Figure 4B). The above results demonstrated that macrophages may play an important role in the development and progression of osteosarcoma. The visualized violin plot was also constructed to prove the above findings (Figure 4C). The PCA of Figure 4D showed nothing individual difference between primary and metastasis samples.

### Protein–Protein Interaction Network Construction and Module Analysis

**FIGURE 5:**
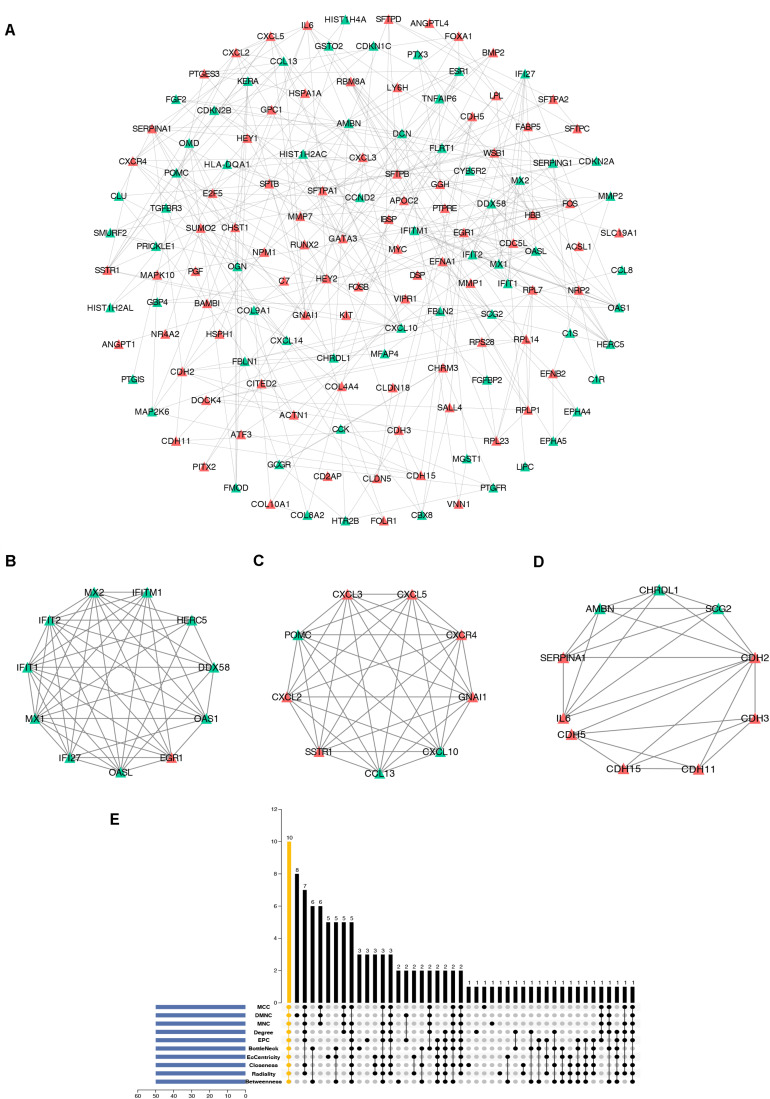
Construction of PPI network, analysis of key modules, and identification of hub genes. **(A)** The whole PPI network. Upregulated genes are marked in red, while the downregulated genes are marked in green. **(B)** PPI network of module 1. **(C)** PPI network of module 2. **(D)** PPI network of module 3. **(E)** Hub genes were identified by intersection of 50 genes from 10 algorithms including MCC, DMNC, MNC, Degree, EPC, BottleNeck, EcCentricity, Closeness, Radiality, and Betweenness. PPI, protein–protein interaction.

To further study the interaction of robust DEGs, we constructed the PPI network by STRING database. With the confidence >0.9 and hiding the disconnected nodes, a visualized PPI network was created by Cytoscape (Figure 5A). In the final network, there were 148 nodes and 302 edges, including 84 upregulated and 64 downregulated genes. By using MCODE plugin, three key modules were screened from the whole network (Figures 5B–D). The robust DEGs in module 1 were mainly enriched in type I interferon signaling pathway. BP of genes in module 2 was particularly enriched in chemokine-mediated signaling pathway. In addition, genes in module 3 were mainly enriched in cell–cell adhesion mediated by cadherin, cell–cell junction assembly, and adherens junction organization (Supplementary Table S4).

### Hub Gene Identification

cytoHubba is a Cytoscape plugin that allows the use several topological analysis algorithms, including MCC, DMNC, MNC, Degree, EPC, BottleNeck, EcCentricity, Closeness, Radiality, Betweenness, and Stress. These approaches can be used to predict and explore important nodes in PPI networks. Scores from topological algorithms are assigned to each node in a PPI network. According to the gene score, the top ranked genes can be considered as the hub genes. In the present study, we used 10 topological analysis algorithms (MCC, DMNC, MNC, Degree, EPC, BottleNeck, EcCentricity, Closeness, Radiality, and Betweenness) to rank the top 50 genes of the whole network. The intersection of these 50 genes from the 10 algorithms revealed the 10 hub genes: *POMC*, *EGR1*, *CXCL10*, *SERPINA1*, *OAS1*, *MYC*, *CXCR4*, *CXCL2*, *CHRDL1*, and *GNAI1* (Figure 5E). The description of the 10 hub genes is shown in Table 1, including full names, synonyms and primary functions.

**TABLE 1:** Description of the 10 hub genes.

Gene	Full name	Synonyms	Function
POMC	Pro-opiomelanocortin		Regulation of cytokine-mediated pathway and signal transduction
EGR1	Early growth response protein 1	KROX24, ZNF225	Transcriptional regulator. Regulation of cell survival, proliferation and cell death
CXCL10	C-X-C motif chemokine 10	INP10, SCYB10	Pro-inflammatory cytokine that is involved in a wide variety of processes such as chemotaxis, differentiation, and activation of peripheral immune cells
SERPINA1	Alpha-1 antitrypsin	AAT, PI	Inhibitor of serine proteases
OAS1	2′-5′-oligoadenylate synthase 1	O1AS	Regulation of interferon-gamma-mediated pathway
MYC	Myc proto-oncogene protein	BHLHE39	Activating the transcription of growth-related genes
CXCR4	C-X-C chemokine receptor type 4		Enhancing MAPK1/MAPK3 activation and involving in the AKT signaling cascade
CXCL2	C-X-C motif chemokine 2	GRO2, GROB, MIP2A, SCYB2	Chemokine-mediated signaling pathway
CHRDL1	Chordin-like protein 1	NRLN1	Cell differentiation and negative regulation of BMP signaling pathway
GNAI1	Guanine nucleotide-binding protein G(i) subunit alpha-1		GTPase activity and regulation of cell cycle and cell division

### Survival Analysis

**FIGURE 6:**
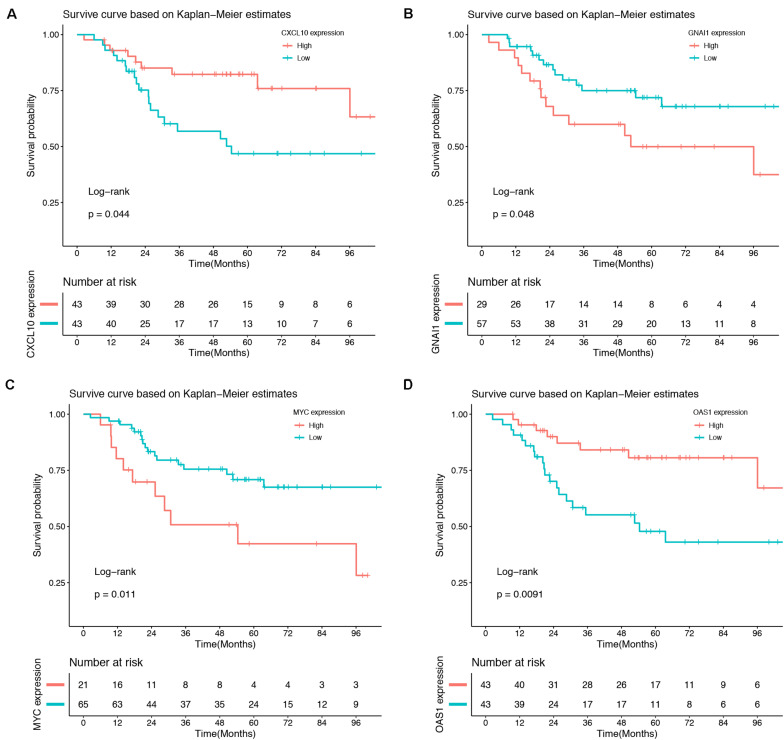
Survival analysis. Gene changes of *CXCL10* **(A)**, *GNAI1* **(B)**, *MYC* **(C)**, and *OAS1* **(D)** were significantly correlated with the overall survival of osteosarcoma patients (*P* < 0.05).

Association between 10 hub genes and the overall survival of osteosarcoma patient were analyzed using R package. Based on each hub gene’s best-separation cutoff value, osteosarcoma patients’ samples within the TARGET-osteosarcoma dataset were divided into two groups to get the Kaplan–Meier (K-M) survival curves. The results demonstrated that gene changes of *CXCL10* (*P* = 0.044), *GNAI1* (*P* = 0.048), *MYC* (*P* = 0.011), and *OAS1* (*P* = 0.0091) were significantly correlated with the overall survival of osteosarcoma patients (Figure 6).

## Discussion

More and more research based on public database such as GEO and TARGET database has been done to determine biomarkers in osteosarcoma. For example, Wang et al. (20) used the microarray data of 42 different age groups (<20- and >20-year-old) osteosarcoma samples from GSE39058 to find 2113 DEGs, including 1476 upregulated and 637 downregulated genes. Similarly, they also identified 15 differentially expressed miRNAs (DEMs) in GSE39040, and functional enrichment analysis showed that upregulated DEMs were mainly enriched in cell growth and response to growth factor, and downregulated DEMs were involved in cytokine receptor activity. Moreover, using GEO database, Dai et al. (21) screened candidate genes for predicting the response to chemoresistance in osteosarcoma by miRNA–mRNA network. However, the differentially selected genes in these studies are all based on a single dataset, and the small size of samples will cause the instability of results. We integrated three datasets using RRA method, which is standard and robust, compared with other studies on osteosarcoma.

In our study, a total of 524 robust DEGs were determined by RRA method, including 272 upregulated and 252 downregulated genes. The results of GO and KEGG pathway enrichment analyses indicated that the robust DEGs were significantly correlated with ECM organization, cell adhesion molecule binding, cell–cell adherens junction, collagen-containing ECM, and TGF-beta signaling pathway, which were associated with tumorigenesis and metastasis. Through immune cell infiltration analysis, we compared the infiltration of immune cells in primary and metastatic osteosarcoma specimens. We also constructed the PPI network by STRING database and module analysis; finally, we screened 10 hub genes by cytoHubba including *POMC*, *EGR1*, *CXCL10*, *SERPINA1*, *OAS1*, *MYC*, *CXCR4*, *CXCL2*, *CHRDL1*, and *GNAI1*. Survival analysis of hub genes based on the TARGET database was also performed in our study.

Based on the results of enrichment analyses, the GO terms and KEGG pathways were explored in osteosarcoma. A substantial body of studies indicated that epithelial-to-mesenchymal transition (EMT) is a process needed for metastasis, during which the loss of cell–cell junction such as adherens junctions, allows tumor cells dissociate from the primary site and acquire the motility to invade stroma (22–25). EMT is also reported to confer resistance to anoikis, which is necessary to survive in the circulation (26). In addition to the involvement of EMT process, the tumor-related ECM is also a key factor in tumor progression. In fact, ECM re-organization such as collagen deposition mediated by collagen-binding integrins may be a general cue for prognosis of tumors (27). Consistent with the above conclusions, the results of GO enrichment analysis, such as ECM organization, cell adhesion molecule binding, cell–cell adherens junction, and collagen-containing ECM, indicate their involvement in the progression of osteosarcoma. Additionally, enrichment of robust DEGs in some KEGG pathways, including TGF-beta pathway and wnt pathway, also demonstrates their relationship with osteosarcoma development. The overexpression of TGF-βs is related with the presence of lung metastasis (28) and is associated with high-grade osteosarcoma (29). Inhibition of wnt pathway can reduce osteosarcoma invasiveness by reversing the EMT (30). On the basis of the above results, we showed that the robust DEGs were highly associated with pathogenesis and progression of osteosarcoma. Furthermore, on the basis of the analysis of modules, we found that three key modules are mainly related to type I interferon signaling pathway, chemokine-mediated signaling pathway, and cell–cell adhesion functions. In the type I interferon pathway, interferon-α has been widely studied. Interferon-α reportedly enhanced the apoptosis of osteosarcoma cells mediated by etoposide (31) and doxorubicin (32). Whether the weakening of interferon signaling pathway plays an important role in the development and metastasis of osteosarcoma deserves further study. It has been reported that chemokine-mediated pathways induce the metastasis of primary tumors to distant target organs. In osteosarcoma, the interaction between chemokine CXCL12 and its receptor CXCR4 drives the metastasis of osteosarcoma cells to the lung (33). CXCR3 and its ligands have the same role in promoting lung metastasis of osteosarcoma (34). In addition, the CXCR7 receptor promotes osteosarcoma lung metastasis (35) and has been recognized as a second receptor with high affinity to CXCL12 (36). The interaction of CXCR7/CXCL12 in the progression of osteosarcoma needs to be further investigated.

In the past few decades, accumulating evidence has indicated that cancer initiation and progression are related with not only cancer itself but also tumor microenvironment (TME) (37, 38). TME is a complex including ECM, exosomes, and stromal cells (39). Among stromal cells, tumor-associated macrophages (TAMs), namely, the M2 type macrophages, have been reported to promote angiogenesis, matrix remodeling (40) and are closely associated with osteosarcoma progression and prognosis (41). In our study, we found that macrophages are the most principal infiltrating immune cells in osteosarcoma including undifferentiated macrophage M0 and macrophage M2; thus, the role of macrophages, especially M2 type macrophages, in the microenvironment of osteosarcoma needs to be further clarified.

Based on the PPI network construction, 10 hub genes were identified. Among these hub genes, six key genes were screened to explore their roles. Serpin peptidase inhibitor clade A member 1 (SERPINA1), a protease inhibitor, was reported to be a predictor in breast cancer (42) and colorectal cancer (43). However, the diagnostic and prognostic roles of SERPINA1 in osteosarcoma were still obscure. Chemokine CXCL2 was reported to be related with prognosis of bladder cancer (44), but its role in the progression of osteosarcoma was still unclear. Early growth response protein 1 (EGR1), a zinc-finger transcription factor, was reported to be involved in cell proliferation and migration (45, 46). And an increasing number of studies have shown that EGR1 is highly associated with cancer development and progression. Liu et al. reported that EGR1 was essential for HNF1A-AS1-mediated cell growth and invasion of gastric cells (47). EGR1 was also reported to promote prostate cancer bone and brain metastasis, as demonstrated by the reduction of blood vessel density in brain and bone caused by decreased EGR1 expression (48). In our study, the expression of EGR1 was higher in metastatic osteosarcoma tissues than primary tissues, but its role in osteosarcoma remained unclear. C-X-C motif ligand 10 (CXCL10) is a member of the CXC subfamily of chemokines and acts through CXC receptor 3 (CXCR3) (49, 50). The prognostic role of serum CXCL10 was proved by Yu et al. in colorectal cancer, and the authors also indicated that the high levels of serum CXCL10 were highly related with liver metastasis (51). Similarly, a study was also reported that high circulating levels of CXCL10 are a biomarker for worse survival in osteosarcoma (52). According to our result, high expression of CXCL10 in osteosarcoma tissues predicted a better survival. The differences in the above conclusions may be due to the different sources of CXCL10; the association between the CXCL10 expression in tissues and overall survival of osteosarcoma patients need to be further studied. Genetic mutations of tumor suppressor such as *TP53* and *RB1* are highly associated with osteosarcoma development (53, 54). In addition, the mis-regulated expression of oncogene *MYC* is often found in osteosarcoma patients (55). A study indicated that the overexpression of *c-myc* promoted osteosarcoma cells invasion through MEK-ERK pathway (56). Consistent with above result, the expression of *MYC* was higher in metastatic osteosarcoma tissues compared with the primary tissues, and the low expression of *MYC* predicted a better overall survival in our study. In a word, the *MYC* expression may act as a biomarker in osteosarcoma metastasis. Chemokine receptor 4 (CXCR4), a seven-transmembrane G protein, has been implicated to mediate the metastasis of several tumors and has become a potential target for tumor therapy (57). Several studies highlighted that the overexpression of CXCR4 potentiated osteosarcoma growth and lung metastasis (58–60). The CXCR4 antibody (61) and antagonist AMD3100 (62) were reported to suppress osteosarcoma cell invasion and lung metastasis, and it revealed that CXCR4 may act as a therapeutic agent to inhibit osteosarcoma progression.

## Conclusion

In conclusion, by using integrated bioinformatics analysis such as RRA method, we identified the significant robust DEGs and gene modules in osteosarcoma. The enrichment analyses of DEGs showed that they were closely associated with osteosarcoma development and progression. We not only identified immune cell infiltration in osteosarcoma tissues, but we also screened 10 hub genes. After the above discussion, we found that genes *EGR1*, *CXCL10*, *MYC*, and *CXCR4* may be considered as novel biomarkers of osteosarcoma, and more studies need to be done to illuminate their contribution in the diagnosis and prognosis of osteosarcoma.

## Data Availability Statement

The datasets presented in this study can be found in online repositories. The names of the repository/repositories and accession number(s) can be found in the article/Supplementary Material.

## Author Contributions

TY and JN conceived the study and thoroughly revised the manuscript. JN, WG, WW, ZZ, TR, and YH acquired and analyzed the data. JN, HZ, YY, and XL wrote the manuscript. All authors approved the final version of the manuscript.

## Conflict of Interest

The authors declare that the research was conducted in the absence of any commercial or financial relationships that could be construed as a potential conflict of interest.

## Footnotes

1 https://www.ncbi.nlm.nih.gov/geo/

2 http://www.string-db.org/

3 https://cytoscape.org

4 https://ocg.cancer.gov/programs/target
